# Room-temperature NH_3_ sensing of graphene oxide film and its enhanced response on the laser-textured silicon

**DOI:** 10.1038/s41598-017-15270-3

**Published:** 2017-11-07

**Authors:** Suwan Zhu, Haibin Sun, Xiaolong Liu, Jun Zhuang, Li Zhao

**Affiliations:** 1 Collaborative Innovation Center of Advanced Microstructures, State Key Laboratory of Surface Physics and Department of Physics, Fudan University, Shanghai, 200433 China; 2 Shanghai Ultra-Precision Optical Manufacturing Engineering Center and Department of Optical Science and Engineering, Fudan University, Shanghai, 200433 China

## Abstract

Electricity-based response to NH_3_ of graphene oxide (GO) is demonstrated at ppm level at room temperature. The GO film prepared on planar silicon substrate shows weak response when exposed to 50 ppm NH_3_, response time less than 30 s and recovery time about 100 s. More interestingly, the GO film coated on laser-textured silicon substrate shows significant enhancement for sensor response, and meanwhile response/recovery time is mainly preserved. Furthermore, a good response of textured GO film is detected in a dynamic range of 5–100 ppm NH_3_. The surface morphology and chemical bonds of the textured GO film are studied by scanning electron microscope (SEM), Fourier Transform Infrared (FT-IR) Spectrometer and X-ray Photoelectron Spectrometer (XPS), respectively. The NH_3_ response is attributed to the polar oxygen configurations of GO and the enhanced response is due to the richer oxygen configurations that stem from cobwebby microstructure of GO.

## Introduction

Recently, the properties of graphene-related materials have aroused worldwide interests due to their attractive mechanical properties, electrical properties and potential applications^1–4^. Various graphene-based materials have been prepared for potential applications such as electronics, catalysis, energy storage, gas sorption, storage, separation and sensing^5^. One promising application for the graphene-based devices is gas sensor. Compared with the traditional sensing materials such as semiconducting metal oxide^6,7^ or porous silicon^8,9^, which are usually associated with high power consumption, high operating temperature, and slow response/recovery time, graphene-based materials show potentials in overcoming their shortages due to the superior properties like high electric conduction and large surface area. Moreover, the high-quality two-dimensional crystal structures would render it possible to detect minuscule changes in exposure to a small number of gas molecules.

Encouraged by the properties mentioned above, many works have followed to investigate practical solutions to achieve gas detection. Theoretical aspects of the molecular adsorption on graphene oxide are reported^10^. The first-principle study suggests that GO shows a better performance than graphene for NH_3_ detection due to the fact that the surface-active defect sites (epoxy and hydroxyl groups) of GO that promote the interactions with NH_3_ molecules^11^. In view of experiments, Prezioso *et al*. observes that the GO flakes showed a typical p-type response by testing the device in both reducing and oxidizing environments, whereas its gas sensing behaviors are not ideal owing to insignificant change in electrical transport properties^12^. To improve the gas response, partially reduced graphene oxide has then been considered widely through chemical and thermal reduction^13–15^. However, pristine GO for gas sensing especially for NH_3_ is rarely reported^16,17^.

In this paper, the sensing properties of GO for NH_3_ is studied, and the electricity-based response at ppm level is observed at room temperature (RT). Besides the planar silicon substrate, we also prepare GO film on the unique textured substrate which is fabricated by femtosecond laser (fs-laser) pulses irradiation in SF_6_ gas ambient as reported in our previous works^18^. Such GO film with the special structure resulting from the substrate exhibits a greatly enhanced response compared with the planar one. To understand the mechanism underlying all the sensing behaviors, the details such as microstructure of the samples and chemical bonds of GO are investigated.

## Materials and Methods

### Fabrication of GO sensors

**Figure 1:**
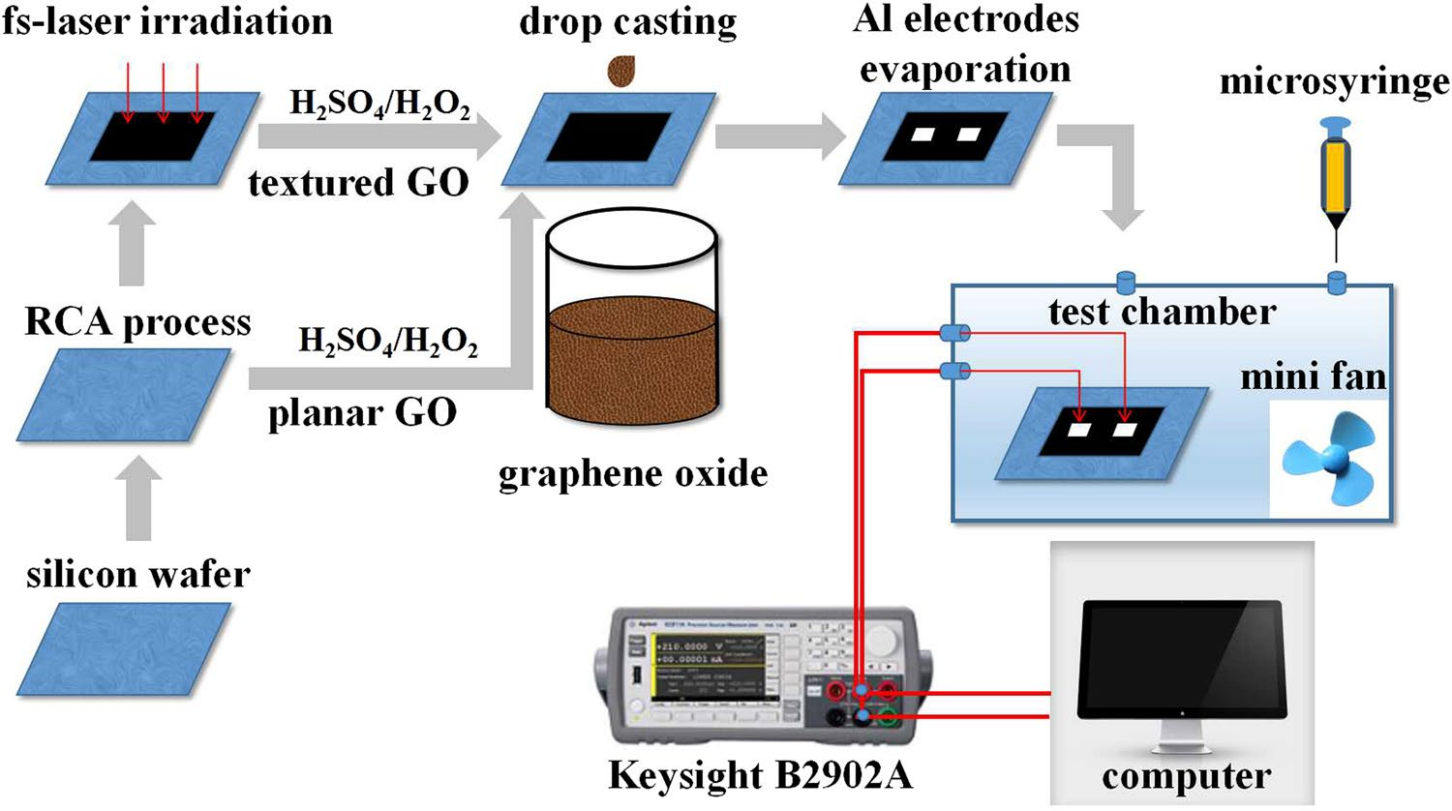
Schematic diagram of sensors preparation and gas sensing test system.

A commercially available aqueous colloidal suspension containing 2 mg/ml GO (flake size with 0.5–5 μm average size and 1.0 ± 0.2 nm thickness) is used. The planar substrate-based GO (pGO) sensor with a 10 × 5 mm^2^ sensing area is prepared directly by depositing 20 μL aqueous colloidal suspension of GO on a flat silicon substrate through drop-casting and then N_2_-dried at RT. A pair of aluminum contacts (3 × 3 mm^2^, 400 nm thickness) is obtained by thermal evaporation onto the surface. To prepare the textured substrate, double-polished p-type Si (100) wafer (1–3 Ω•cm, 250 μm thickness) is cleaned by standard RCA (Ratio Corporation of America) process, and then placed in a stainless-steel vacuum chamber before it is backfilled with SF_6_ at 70 kPa. The wafer is irradiated at normal incidence with a Yb:KGW fs-laser (1 kHz train of 190 fs, 515 nm laser pulses) at a fluence of 8 kJ/m^2^. The laser beam is focused on the sample with a 250 mm focal length lens. Textured silicon with an area of 10 × 5 mm^2^ is formed from translating the silicon wafer by stepper motors in continuous raster scan pattern at a speed of 0.5 mm/s. After the preparation of textured silicon surface, GO and aluminum contacts are then deposited by the same procedures as those for pGO, to obtain textured-substrate-based GO (tGO) sensor. The electrode structure and the size of tGO is the same as those of pGO. Both the silicon substrates are treated with a H_2_SO_4_/H_2_O_2_ solution (7:3) for improving their hydrophilicity before film preparation. The schematic diagram of sensor fabrication is shown in Fig. 1.

### Sensing test and characterization

For measuring the sensing behavior of the sensors in NH_3_ gas, a gas sensing test system is used as shown in Fig. 1, in which the devices are placed in a sealed organic glass test chamber in a constant RT/RH laboratory. The test chamber is filled with clean air before sensing measurement. The sensors’ contacts are made an electrical connection with two silver test probes. A stirring mini-fan inside the chamber is used to promote the uniformity of the gas mixture. The NH_3_ concentration is calculated from the volume ratio of pure NH_3_ to test chamber according to the static volumetric method in which a predetermined amount of pure gas is injected into the chamber directly by a micro-syringe to get the desired concentration^19^. I-V characteristic and dynamic resistance of the sensors during gas injection are recorded by using a precision measurement unit (Keysight B2902A) which is connected to a computer. All the sensing experiments are conducted in the dark in order to reduce the background current. Surface morphology of the laser-textured substrate and GO film is observed by a field emission scanning electron microscope (SEM, Carl Zeiss ΣIGMA500) with an accelerating voltage at 5.0 kV. The chemical species of tGO are studied by Fourier Transform Infrared Spectrometer (FT-IR, Bruker Optics ALPHA) and X-ray Photoelectron Spectrometer (XPS, PHI 5000 C), respectively.

## Results and Discussion

### Micrograph of the substrate and GO film

**Figure 2:**
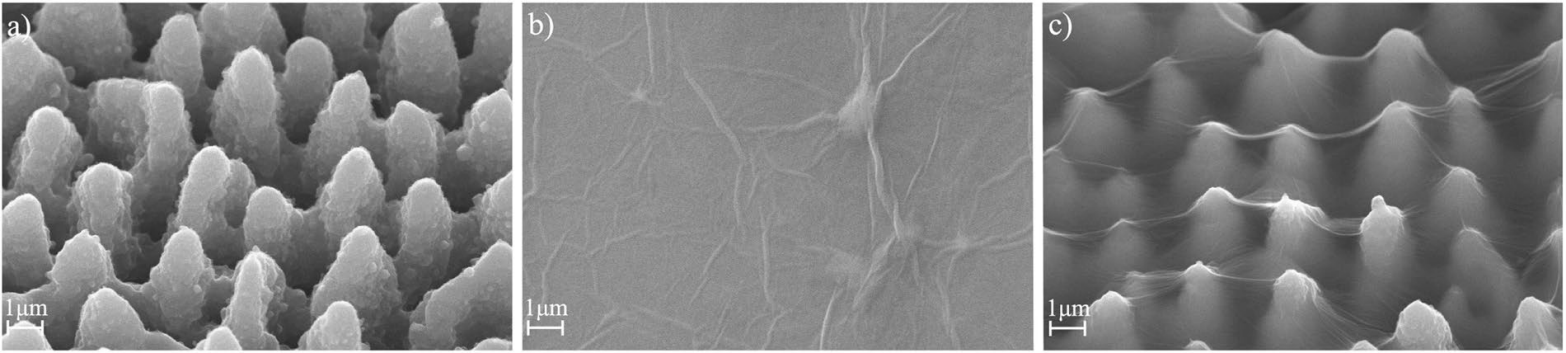
SEM images (viewed at 45°) of: (**a**) laser-textured substrate, (**b**) pGO, (**c**) tGO.

After fs-laser ablation, the morphology of the textured silicon surface is observed by SEM. As shown in Fig. 2(a), conical spikes with an average height of about 5 μm are formed. Figure 2(b) is the micrograph of pGO and Fig. 2(c) shows the morphology of tGO. After deposited on the laser-textured substrate, a unique cobwebby GO film is hung over the top of the spike-like substrate (see Fig. 2(c)) rather than adsorbed on the spike’s surface.

### I-V characteristics of the suspended GO

**Figure 3:**
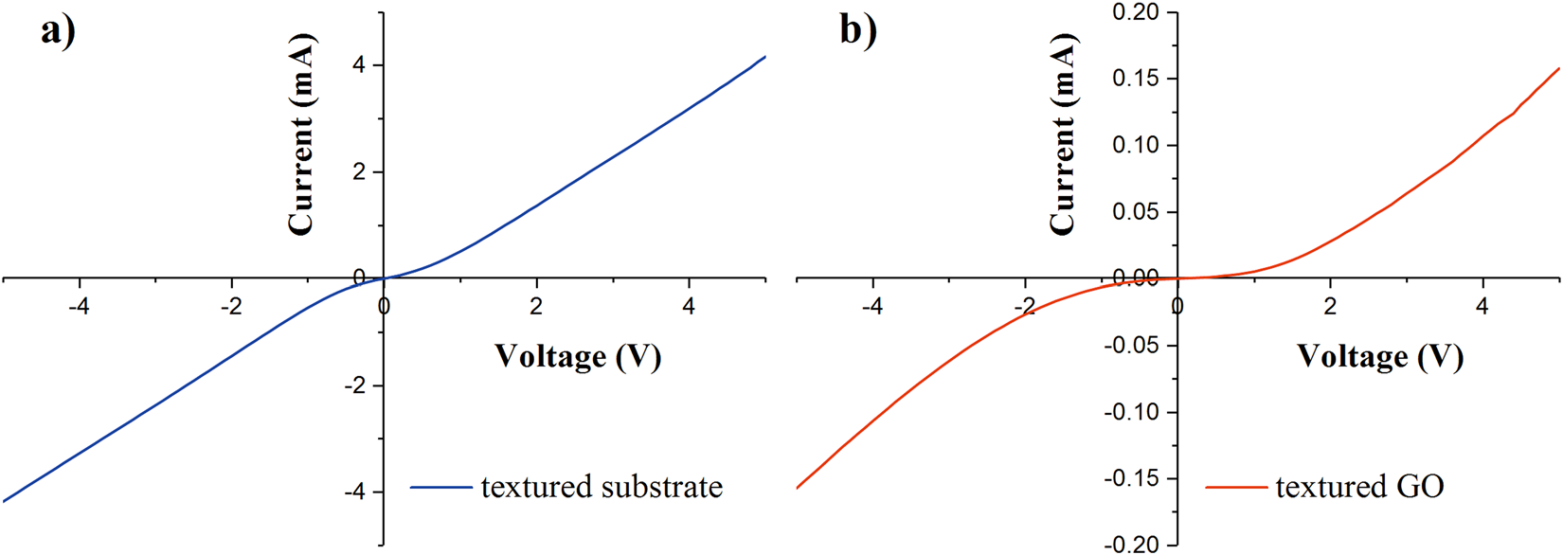
I-V characteristics of the textured substrate and tGO.

In order to see whether the charge transport is sufficiently isolated from the suspended GO film to the silicon substrate, I-V characteristics of the substrate and tGO are measured, as seen in Fig. 3. Either sample shows a non-linear I-V characteristic from −5 V to 5 V bias. The maximum current under 5 V bias is greater than 4 mA for the bare substrate (see Fig. 3(a)), while the corresponding value is less than 0.17 mA for tGO, which is approximately one-twentieth of the value for the silicon substrate (see Fig. 3(b)). The electrical difference indicates that tGO over the spike-pillar structure is adequately insulated from the substrate.

### NH_3_ response of the sensors

**Figure 4:**
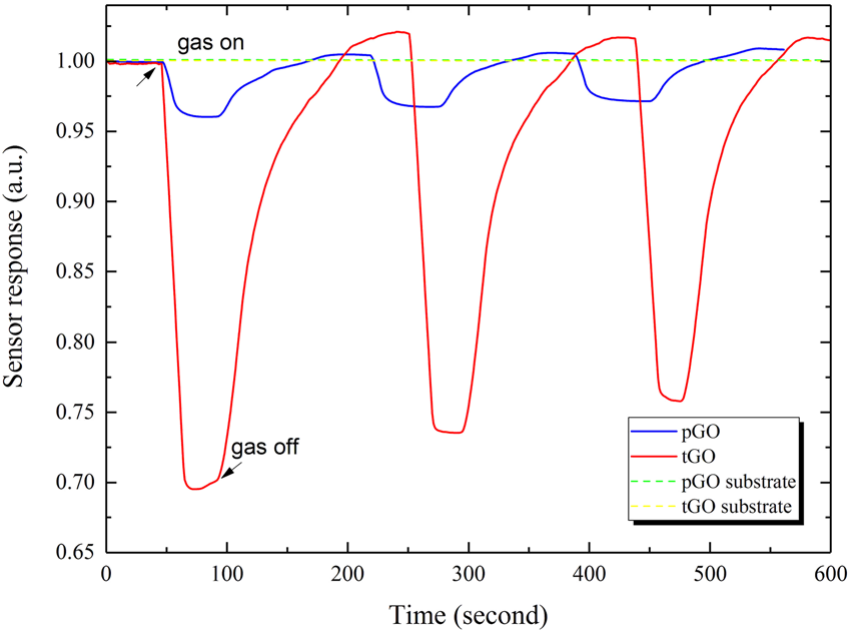
Sensing behaviors of pGO and tGO for three consecutive cycles of response and recovery at 50 ppm NH_3_.

Gas response of the two sensors is measured at RT with a RH of ~40%. The sensor’s resistance is obtained directly between the two contacts taken from the top of the GO film. The normalized response curve is obtained from the dynamic resistance divided by the initial value in air. As the blue curve in Fig. 4 shows, response time and recovery time of pGO is less than 30 s and 100 s at 50 ppm concentration, respectively. Nevertheless, the sensor response is relatively weak: a decline in resistance from 1 down to 0.96. The sensing behavior of tGO at the same gas concentration is displayed by the red curve in Fig. 4. By contrast, the sensor response of tGO declines to ~0.75 with a significant enhancement, meanwhile response time and recovery time is slightly prolonged. Three consecutive cycles of gas sensing process are performed to check the response reproducibility of both two sensors. It is worth mentioning that the sensing behavior of bare substrate with a similar setup without GO are carried out in the meantime. The green and yellow dotted curve in Fig. 4 represents the gas response of bare substrate of pGO and tGO, respectively. No significant gas response is observed for pristine substrate, which means the dynamic response signals positively result from the GO material.

**Figure 5:**
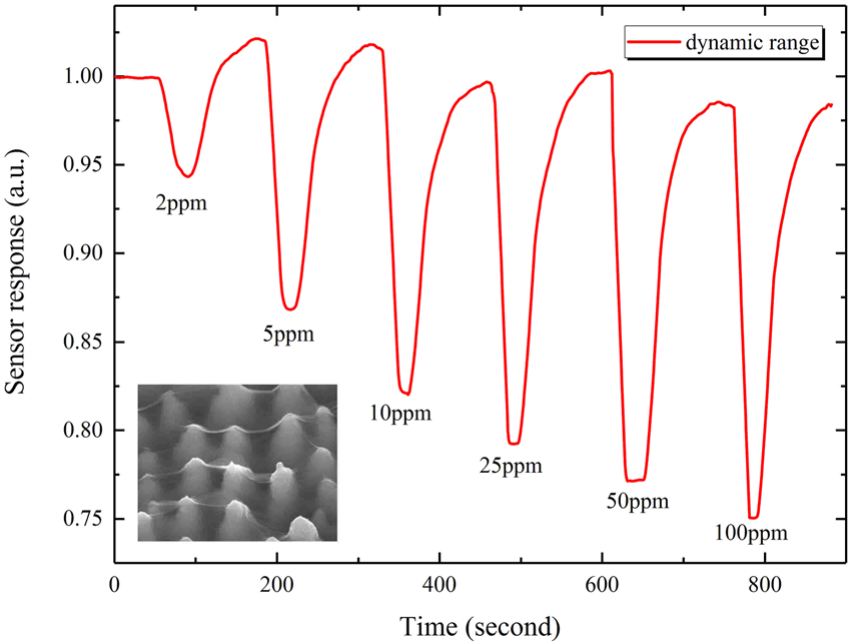
Sensing response of tGO in a dynamic range of 2 to 100 ppm NH_3_.

The sensor response of tGO in a dynamic range of 2–100 ppm NH_3_ is demonstrated in Fig. 5. It can be seen that tGO shows a significant NH_3_ response with ~0.94 even at a concentration as low as 2 ppm, as well as in a higher level (100 ppm) with ~0.7. The baseline shift in Figs 4, 5 probably indicates an irreversible interaction of NH_3_ molecules with partial adsorption sites on GO at RT, which is confirmed by the subsequent FT-IR and XPS spectra.

### Sensing performance under different drops volume

**Figure 6:**
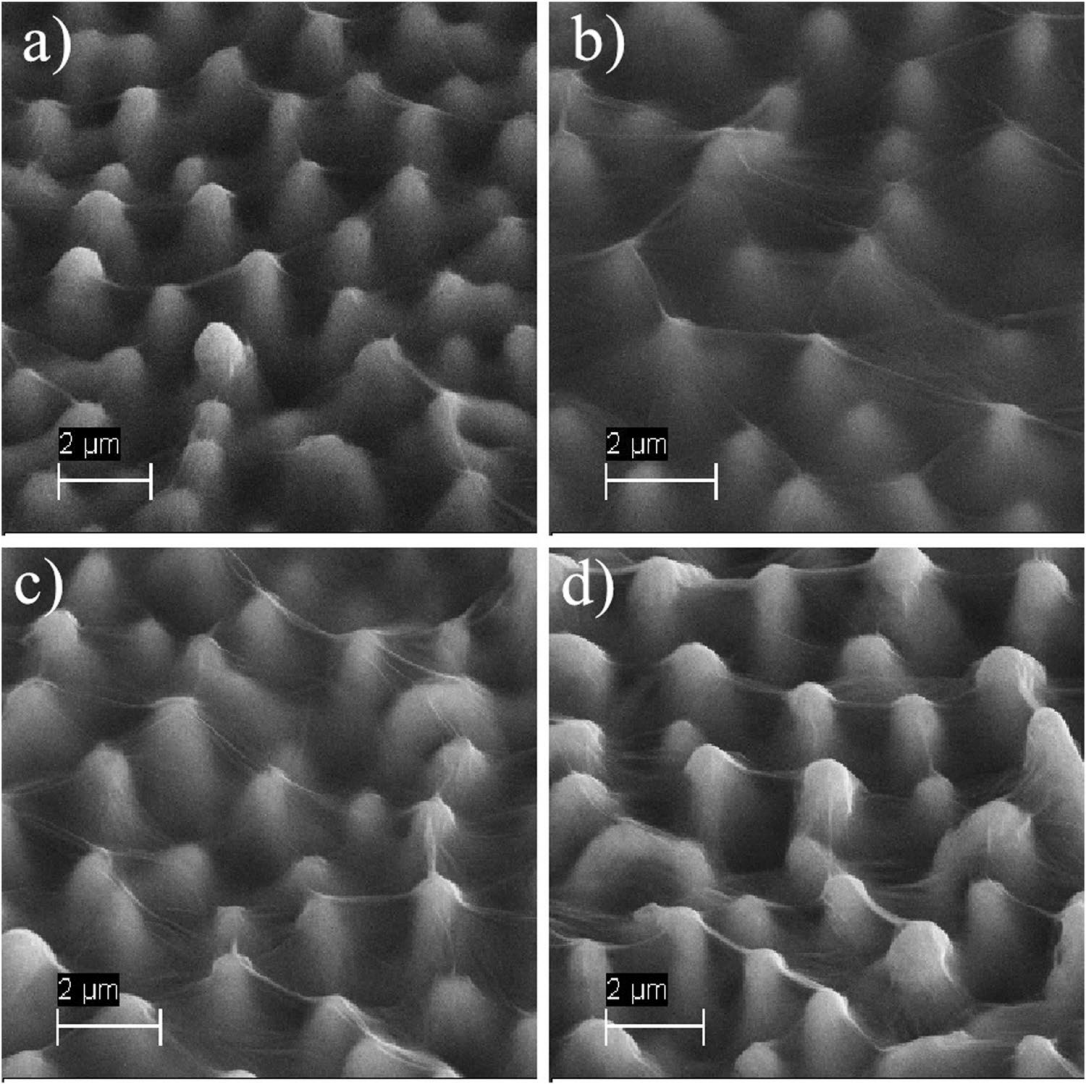
SEM images of tGO film prepared by different drops volume.

**Figure 7:**
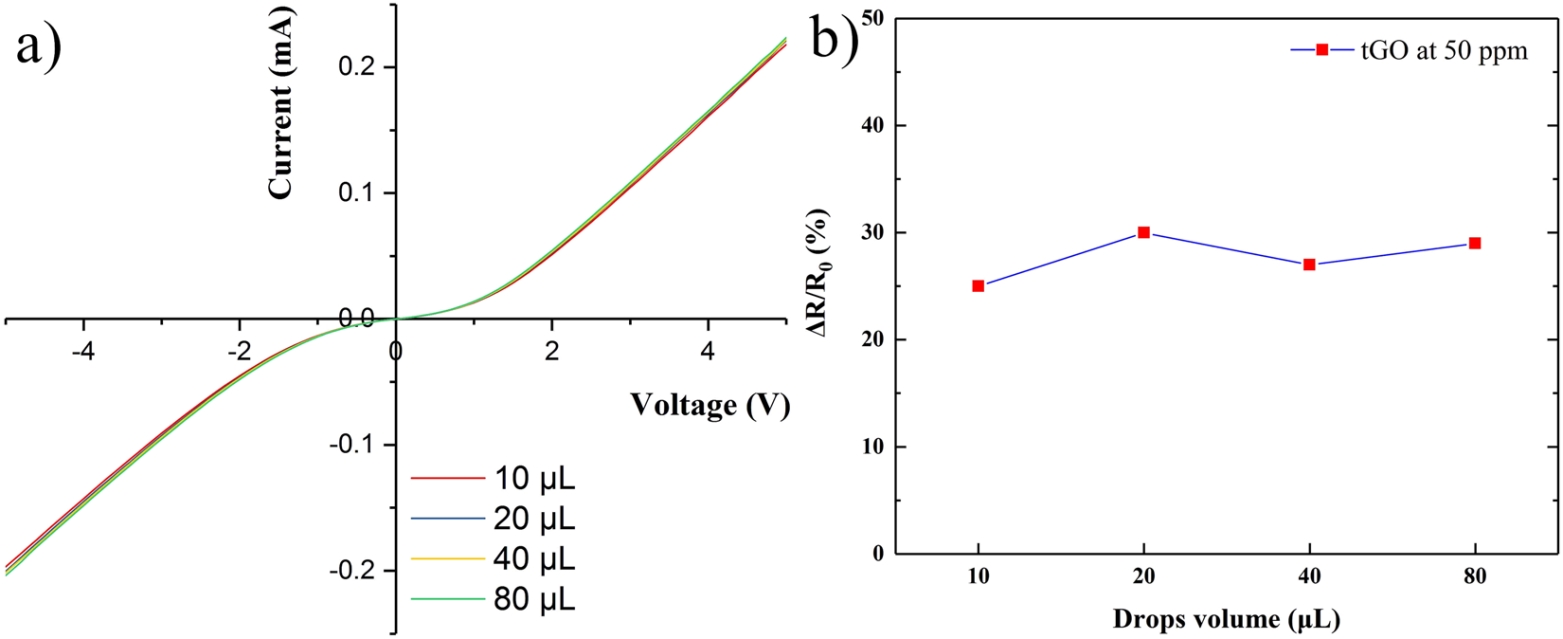
Characteristics of tGO sensor from different drops volumes: (**a**) I-V curves, (**b**) relative response.

In order to investigate the effect of different GO drops volume on sensing performance, a contrast test is carried out. In this test, I-V characteristics and sensor responses for tGO are measured under drops volume of 10, 20, 40 and 80 μL GO solution, respectively. The concentration of NH_3_ is set at 50 ppm and the textured substrate is the same as that from Fig. 2(a). Figure 6 represents the surface morphology of the four samples. The differences among these tGO films are indistinguishable by SEM images. In Fig. 7, it can be observed that the samples show a highly consistent performance on both I-V characteristic and sensing response. The I-V curves show the electrical consistency for the four samples in Fig. 7(a). Besides, the relative responses ΔR/R_0_ are entirely within the range of 25% to 30% at 50 ppm NH_3_, where ΔR is the variation of sensor resistance during sensing process and R_0_ is the sensor resistance in air. The results reveal that GO layer and sensing performance are not sensitive to drops volum during preparation. In fact, we have also found that only a certain amount of GO solution can be deposited on the silicon substrate during the preparation process, forming a thin film, while the rest drains off the substrate by gravity.

### Gas selectivity of tGO sensor

**Figure 8:**
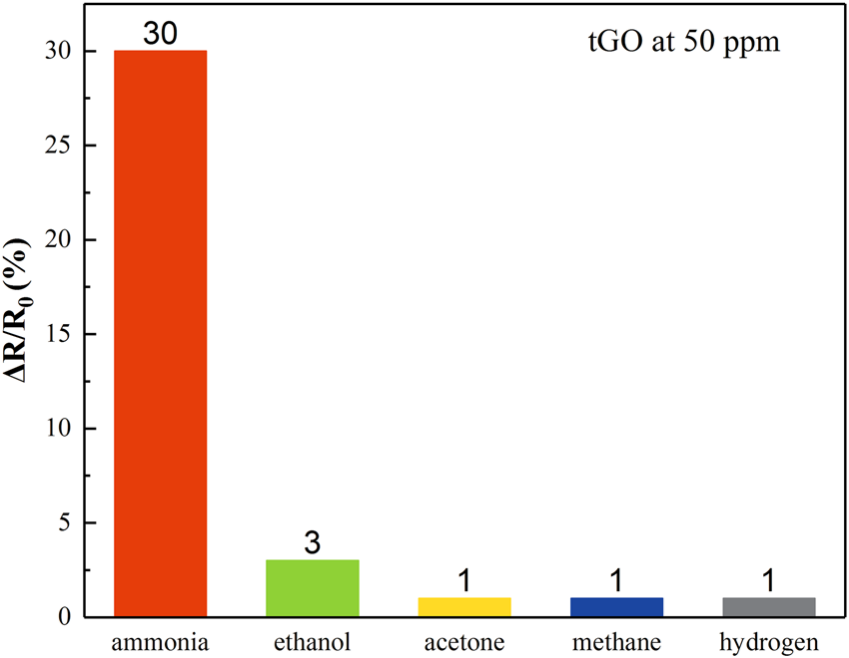
Gas selectivity of tGO at 50 ppm concentration of several different gases.

For tGO sensor, gas responses in other ambient gases like ethanol, acetone, methane and hydrogen have been measured in the same experimental condition. As shown in Fig. 8, only a relative response ΔR/R_0_ of ~3% is observed in ethanol ambient. The sensor exhibits a much higher response to NH_3_ than to other referenced gases, indicating that GO material shows good gas selectivity to NH_3_.

### Sensing mechanism

The results in Fig. 4 show that GO film indeed has response to NH_3_ gas. It is worth noting that pGO film has already shown rapid response and sensible response at RT, which could make it a potential NH_3_ sensing material based on electricity. Compared with pGO device, Fig. 4 also shows that tGO exhibits significant enhancement in sensor response, and meanwhile response/recovery time are slightly lengthened. Besides, it can also be observed that both pGO and tGO sensors’ resistance declines when the devices are in exposure to NH_3_, and this phenomenon is different with the reported results in ref.^17^. The authors find that the resistance of GO material increases when exposed to NH_3_ ambient, and they attribute the phenomenon to a hole depletion mechanism, where GO can be treated as a p-type semiconductor and the absorption of an electron-donating compound such as NH_3_ leads to the increase of sensor resistance. To understand the observed phenomena, the surface details of the two devices are studied by both SEM and FT-IR.

**Figure 9:**
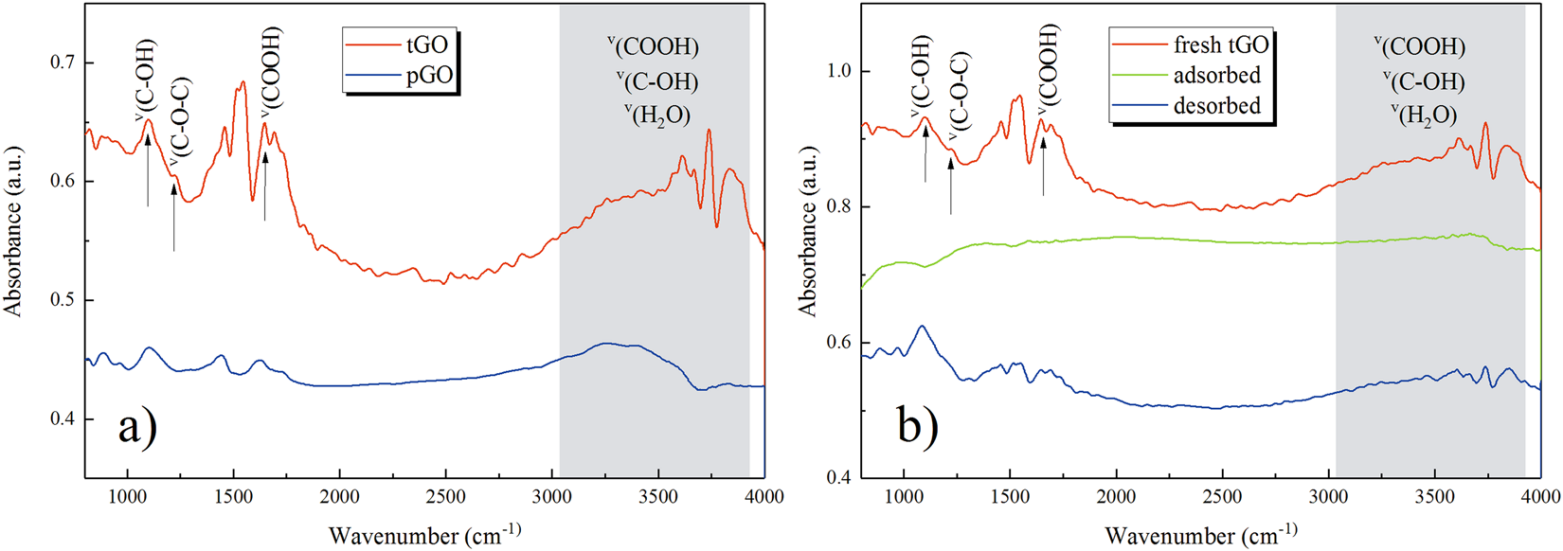
FT-IR spectra of: (**a**) tGO and pGO, (**b**) freshly-prepared, NH_3_-adsorbed and NH_3_-desorbed tGO.

As shown in Fig. 2(c), the tGO film is not just a simple replica of the substrate morphology, its cobwebby profile is different from that of spike-like substrate as well as from the plane’s. In addition, for the pGO sensor in Fig. 2(b), a thin GO film is fit tightly on the surface of the planar substrate. More interestingly, the cobweb-like GO film is just hung over the top of the microstructured substrate. I-V characteristics of bare substrate and tGO film in Fig. 3 have confirmed that tGO film over the spike-pillar structure is adequately insulated from the substrate. Figure 9(a) shows FT-IR spectra of tGO and pGO film. As the red curve depicts, various oxygen configurations are found as those in ref.^20^ for tGO, including the vibration modes of epoxide (C-O-C) (1230–1320 cm^−1^, asymmetric stretching), carboxyl (COOH) (1650–1750 cm^−1^ including C-OH vibrations at 3530 cm^−1^ and 1080 cm^−1^), and hydroxyl (C-OH) (1070 cm^−1^ and 3050–3600 cm^−1^) with all C-OH vibrations from COOH and H_2_O. As the interaction of NH_3_ with graphite oxide studied by Slabaugh *et al*.^21^, GO contains radicals like carboxyl, hydroxyl, epoxy, and possibly other groups attached to the hexagonal platelets. These acidic radicals offer energetic sites for chemical adsorption for polar molecules. Even though GO is electrically insulating due to those oxygen groups, the conductivity can be partially restored by the removal of oxidizing groups using chemical or thermal reduction^22^. Therefore, the possible sensing mechanism is that, when GO is in exposure to reducing gaseous like NH_3_, the electronic charges are transferred to these insulating sites from adsorbed ammonia molecules, leading to partial reduction. This viewpoint is confirmed by FT-IR spectra in Fig. 9(b): the red curve demonstrates the chemical configurations from freshly-prepared tGO. However when the fresh tGO is placed in the gas ambient of 50 ppm NH_3_, the IR-absorbance of these oxygen configurations decreases drastically and almost vanishes, as the green curve shows. More interestingly, such insulating configurations basically recover after NH_3_ desorption, as shown in the blue curve. Therefore, the resistance of the pGO film declines and rises as one full sensing cycle when it is in exposure to NH_3_ gas.

Besides, we have also noticed that recovery of these oxygen configurations is incomplete after NH_3_-desorption, especially for COOH (1650–1750 cm^−1^ including C-OH vibrations at 3530 cm^−1^ and 1080 cm^−1^) and C-OH (1070 cm^−1^ and 3050–3600 cm^−1^), because the IR-absorbance of each species becomes weaker than that of the fresh sample. The subsequent consequence is that both the sensor resistance and response will not recover to its initial state. In Fig. 4 the sensor response toward 50 ppm fixed concentration NH_3_ decreases gradually in three cycles. Besides, Fig. 5 shows that the baseline (resistance) is on a declining trend after three consecutive cycles of gas sensing process. However, the baseline did not decline immediately within a few cycles (three or less) in both Figs 4, 5, this could be attributed to signal instability of the weak reaction between NH_3_ and GO at RT.

**Figure 10:**
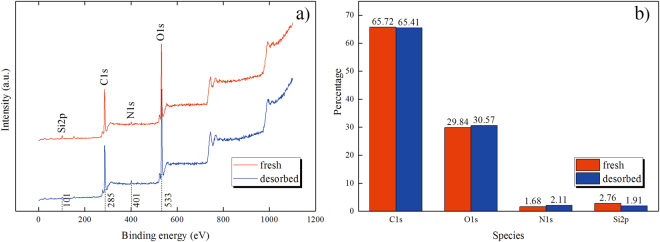
The freshly-prepared and NH_3_-desorbed tGO film: (**a**) XPS spectra, (**b**) the proportion of C 1s, O 1s, N 1s and Si 2p species in each sample.

In addition, XPS spectra have also support a similar conclusion. Figure 10(a) shows the XPS spectra giving the peaks associated with carbon, oxygen, nitrogen and silicon species. In tGO samples, the binding energies of Si 2p, C 1s, N 1s and O 1s are centered at 101, 285, 401 and 533 eV respectively. It can be observed that N 1s component of tGO even after NH_3_ desorption (blue curve) distinctly increases by ~25.6% when compared with that of the fresh one (red curve). Moreover, the variation on the percentage for N 1s is much greater than that of C 1s (0.47%) and O 1s (2.4%), as Fig. 10(b) shows. The decrease of Si 2p, ~30.8%, is probably as a result of the increased nitrogen adsorbed on the tGO surface. These results from FT-IR and XPS spectra undoubtedly reveal the irreversible interaction of NH_3_ molecules with partial adsorption sites on GO at RT.

### Enhanced NH_3_ response

Compared with the pGO film, the tGO film over the spiked substrate has a moderately larger surface area for gas adsorption, as Fig. 2 shows. This is the reason why the tGO device has an enhanced response for NH_3_ sensing: a larger sensing area leads to more effective adsorption sites, resulting in a more significant change of resistance. This explanation is also supported by FT-IR results. As the blue curve shows in Fig. 9(a), similar oxygen groups also occur at the same wavenumbers for pGO, but the intensity of these groups is much weaker than that of tGO, which means that tGO could offer much more acidic adsorption sites for NH_3_ than pGO. Meanwhile, based on the same reason, it is easily imaginable that the response speed should decrease as the observation in Fig. 4. Besides, we don’t rule out any possibility for sensing enhancement in the case when gas diffuses into the back of the tGO film. Figure 5 shows the dynamic range of sensor response at 2 to 100 ppm concentration NH_3_. Notably, the tGO device showes a variable gas response versus different concentration. Along with the increase of gas concentration, the change rate of the sensor response declines. The sensor response at 100 ppm NH_3_ is close to that of 50 ppm, indicating the saturated adsorption is near. As a result, the dynamic range curve suggests that the GO film may be more suitable for NH_3_ detection at a lower concentration level.

In fact, lots of works have tried various methods to increase the gas response of pristine GO material, such as functionalization with additional molecules and nanoparticles^23,24^. Here we present another way to enhance the sensing performance of GO, and this novel technique could be used in combination with the above chemical methods. In other words, it may be suitable for other quasi-two-dimensional graphene-based materials to improve their gas response. Moreover, the results in Fig. 4 imply that the gas sensing properties such as sensor response, detection limit, response and recovery time may be regulated by different film morphologies, which can be easily obtained by changing spike size of the substrate under different laser-ablation conditions. For different application areas, it is obviously useful.

## Conclusions

In summary, we have fabricated two types of NH_3_ sensors based on GO material, the planar-substrate-based and the textured-substrate-based GO sensor. Both the two types of sensors show electricity-based NH_3_ response at RT, indicating that GO material has a potential for detection of ppm-level of NH_3_. Compared with the pGO device, the textured one shows a remarkable enhancement in sensor response at the concentration of 50 ppm NH_3_. Meanwhile, its fast response/recovery time is mainly saved. SEM and I-V results indicate that a cobwebby GO film is uniformly formed on the textured substrate. FT-IR and XPS spectra reveals that the sensing mechanism of GO material is the interaction of oxygen configurations with NH_3._ Moreover, compared with the pGO sensor, such a specific form of GO film could offer more effective molecule adsorption sites for NH_3_, leading to a significant enhancement for gas response. Our preliminary exploration through the textured substrate to get the microstructured GO film may also be suitable for other quasi-two-dimensional graphene-based materials to adjust their gas sensing properties.
